# Arthroscopic Microfracture Technique for Cartilage Damage to the Lateral Condyle of the Tibia

**DOI:** 10.1155/2015/795759

**Published:** 2015-08-05

**Authors:** Hiroyuki Kan, Yuji Arai, Shuji Nakagawa, Hiroaki Inoue, Ginjiro Minami, Kazuya Ikoma, Hiroyoshi Fujiwara, Toshikazu Kubo

**Affiliations:** Department of Orthopaedics, Graduate School of Medical Science, Kyoto Prefectural University of Medicine, Kyoto, Japan

## Abstract

This report describes the use of arthroscopic microfracture to treat a 10-year-old female patient with extensive damage to the cartilage of the lateral condyle of the tibia before epiphyseal closure, resulting in good cartilage recovery. Magnetic resonance imaging showed a defect in part of the load-bearing surface of the articular cartilage of the condyle articular of the tibia. The patient was diagnosed with damage to the lateral condyle cartilage of the tibia following meniscectomy, and arthroscopic surgery was performed. The cartilage defect measured approximately 20 × 20 mm, and microfracture was performed. Arthroscopy performed four months postoperatively showed that the cartilage defect was completely covered with fibrous cartilage, and the patient was allowed to resume sports activities. Four years postoperatively, she has had no recurrence of pain or hydrarthrosis.

## 1. Introduction

Treatment methods for cartilage damage include drilling, microfracture, osteochondral autograft transfer, and autologous chondrocyte implantation. Microfracture promotes the migration of undifferentiated mesenchymal cells from the bone marrow, forming fibrous cartilage tissue to enable recovery. This procedure is simple and less invasive than other treatment methods and yields good results when used to treat cartilage damage to the femur. Only a few reports, however, have assessed outcomes in patients treated with microfracture for damage to the tibial cartilage, and, in those reports, microfracture has been combined with high tibial osteotomy. This report describes the use of arthroscopic microfracture to treat a 10-year-old female patient with extensive damage to the cartilage of the tibial lateral condyle before epiphyseal closure, resulting in good cartilage recovery. The patient and her family were informed that data from her case would be submitted for publication, and they provided their consent.

## 2. Case Presentation

**Figure 1:**
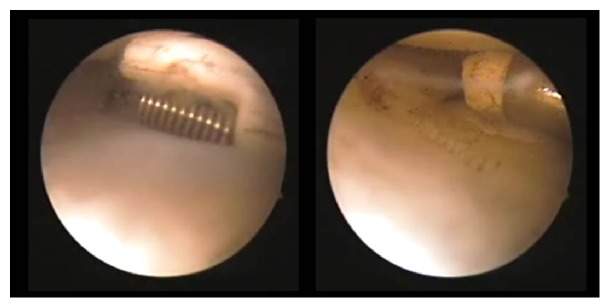
Arthroscopy of both knees was performed, followed by arthroscopic partial meniscectomy with a high-frequency electric knife for both knees.

**Figure 2:**
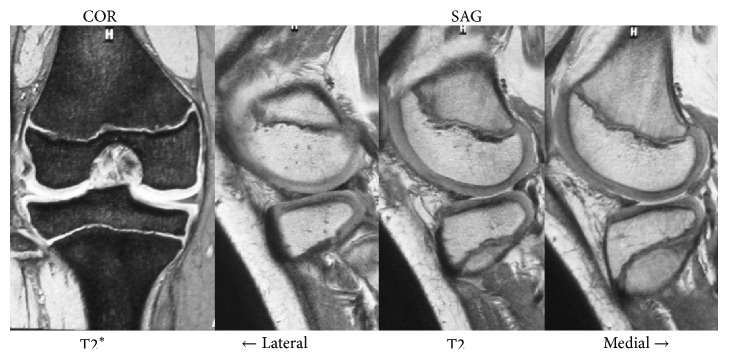
Magnetic resonance imaging (MRI), showing a defect in part of the load-bearing surface of the tibial lateral condyle articular cartilage.

**Figure 3:**
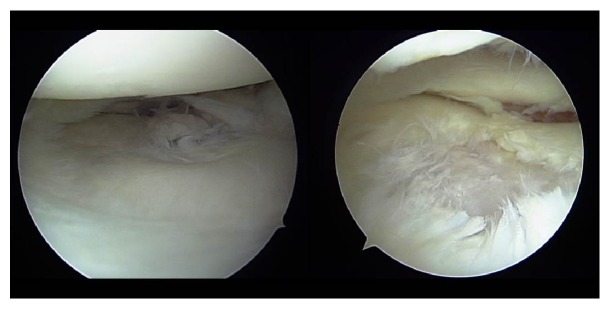
Arthroscopic findings: fibrillation was observed at the margin of the lateral meniscus after resection, and mild fibrillation of the femoral condyle cartilage was also present. A cartilage defect approximately 20 × 20 mm was observed in the load-bearing area of the tibial lateral condyle, and the subchondral bone was exposed.

**Figure 4:**
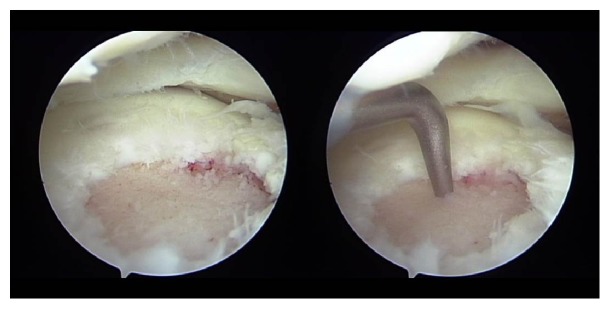
Arthroscopic findings: the damaged cartilage was curetted, and microfracture was performed. An awl with the tip bent 90° was used to perforate the bone at 3 to 4 mm intervals.

**Figure 5:**
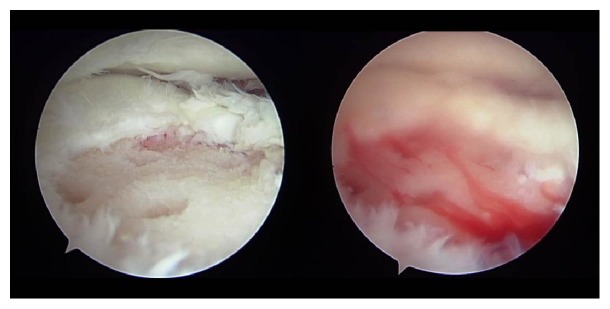
Arthroscopic findings: good hemorrhage from within the bone marrow was confirmed after the tourniquet was released.

**Figure 6:**
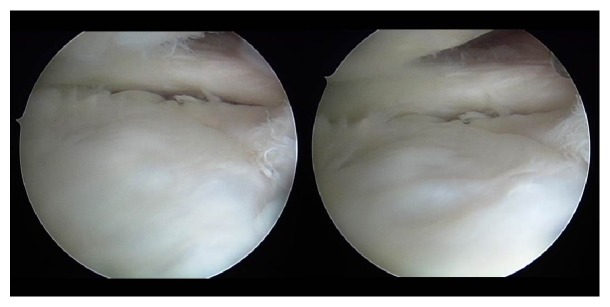
Arthroscopic findings 4 months postoperatively: although probing did reveal softening, the cartilage defect was completely covered with fibrous cartilage.

**Figure 7:**
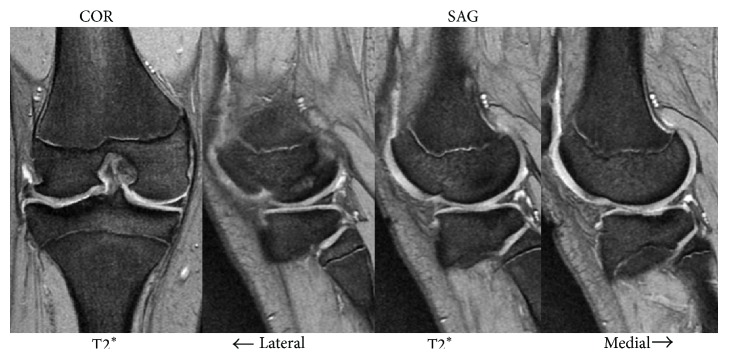
MRI showing that the defect of the tibial lateral condyle articular cartilage was covered with cartilage.

A 10-year-old girl developed left knee pain while jumping rope and was examined at a local clinic. She was diagnosed with complete discoid meniscus of both knees. Both knees were examined arthroscopically, and both underwent arthroscopic partial meniscectomy with a high-frequency electric knife (Figure 1). Two months later, the patient experienced joint pain, swelling, and restricted range of motion in her right knee, and she was referred to our department. Magnetic resonance imaging (MRI) showed a defect in part of the load-bearing surface of the tibial lateral condyle articular cartilage (Figure 2), and arthroscopic surgery was performed. Fibrillation was observed at the margin of the lateral meniscus after resection, along with mild fibrillation of the femoral condyle cartilage. A cartilage defect, measuring approximately 20 × 20 mm, was observed in the load-bearing area of the tibial lateral condyle, and the subchondral bone was exposed (Figure 3). The damaged cartilage was curetted, and microfracture was performed. An awl with its tip bent 90° was used to perforate the bone at 3 to 4 mm intervals (Figure 4), and good hemorrhage from within the bone marrow was confirmed after the tourniquet was released (Figure 5). Postoperatively, the patient completed avoiding weight-bearing for eight weeks, followed by partial weight-bearing until week 12, and full weight-bearing thereafter. Arthroscopy was again performed four months postoperatively. Although probing revealed softening, the cartilage defect was completely covered with fibrous cartilage (Figure 6), and the patient was allowed to restart playing sports. Four years postoperatively, she has experienced no recurrence of pain or hydrarthrosis. Plain X-rays showed no obvious signs of abnormalities, and MRI showed that the defect of the tibial lateral condyle articular cartilage was covered with cartilage (Figure 7).

## 3. Discussion

It is reported that temperature of 45° to 50°C may damage chondrocytes [1, 2]. McCormick et al. reported that potentially damaging temperature elevations can be reached within 1 to 2 seconds near the probe and within 16 seconds at a distance of 5 mm from the probe during continuous radiofrequency (RF) ablation [3]. Therefore, it is highly likely to induce cartilage damage when using an RF in a narrow femorotibial joint. There, articular cartilage is mainly composed of cell-poor extracellular matrix and contains no blood vessels, lymph channels, or nerves. Because its regenerative capacity is poor, articular cartilage repair is limited. Experiments on the knees of ponies showed that small defects, around 3 mm in size, repaired themselves with cartilage tissue, but defects larger than 9 mm did not self-repair [4]. Self-repair of injuries to the articular cartilage of adult humans with cartilage-like tissue is therefore limited to those ≤5 mm in diameter. Moreover, assessments of rat models of cartilage damage showed cartilage repair in young rats, aged 3 to 7 weeks, but no repair with cartilage tissue in adult rats more than 1 year old [5]. Methods of treatment for cartilage damage that will not recover spontaneously include drilling, microfracture, osteochondral autograft transfer, and autologous chondrocyte implantation. A wide range of factors must be considered when choosing a procedure, including the lesion location and area, the patient's age [6], alignment, and level of activity. Treatment guidelines have therefore been established for injuries to the cartilage of the femoral condyle based on the size of the injuries, with microfracture recommended for cartilage injuries <2 cm^2^ in area, osteochondral autograft transfer for those measuring 2–4 cm^2^, and osteochondral autograft transfer or autologous chondrocyte implantation for those with >4 cm^2^ [7–10]. However, no clear criteria have been established for damage to the cartilage of the tibial condyle.

The patient in this report was 10 years old, so epiphyseal closure had not yet occurred. She had developed a large cartilage injury, about 4 cm^2^ in area, on the load-bearing surface of the tibial lateral condyle. This type of injury was unlikely to repair itself; therefore, surgical treatment was indicated. Microfracture was selected because drilling, osteochondral autograft transfer, and autologous chondrocyte implantation all entailed risks of damaging the epiphyseal line. Moreover, for technical reasons, osteochondral autograft transfer and autologous chondrocyte implantation are difficult to perform in load-bearing areas of the tibial lateral condyle. Microfracture promotes the migration of undifferentiated mesenchymal cells from the bone marrow, forming fibrous cartilage tissue to enable recovery. It is a simple procedure and is less invasive than these other methods. Only a few reports have assessed the results of microfracture, combined with high tibial osteotomy to treat injury to the cartilage of the tibial condyle, although good results have been reported when microfracture is used to treat femoral cartilage injury. A study of 72 knees with a mean follow-up period of 11 years reported that both symptoms and function improved [11]. In addition, microfracture showed good results in patients aged <40 years, suggesting that age has an effect on treatment outcomes [12]. The patient described here had extensive damage to the cartilage of the tibial lateral condyle, measuring around 4 cm^2^ in area, but arthroscopy four months postoperatively confirmed good cartilage restoration. Moreover, after 2 years, the patient's clinical course has been satisfactory, indicating that microfracture can be useful even for large cartilage defects of the tibial lateral condyle if epiphyseal closure has not yet occurred.
